# Translating genetic findings to epigenetics: identifying the mechanisms associated with aging after high-radiation exposure on earth and in space

**DOI:** 10.3389/fpubh.2024.1333222

**Published:** 2024-03-22

**Authors:** Nathan A. Ruprecht, Sonalika Singhal, Donald Sens, Sandeep K. Singhal

**Affiliations:** ^1^Department of Biomedical Engineering, University of North Dakota, Grand Forks, ND, United States; ^2^Department of Pathology, University of North Dakota, Grand Forks, ND, United States

**Keywords:** bioinformatics, biological aging, epigenomics, ionizing radiation, rad-age

## Abstract

**Purpose:**

Exposure to radiation is a health concern within and beyond the Earth's atmosphere for aircrew and astronauts in their respective austere environments. The biological effects of radiation exposure from a multiomics standpoint are relatively unexplored and stand to shed light on tailored monitoring and treatment for those in these career fields. To establish a reference variable for genetic damage, biological age seems to be closely associated with the effect of radiation. Following a genetic-based study, this study explores the epigenetic landscape of radiation exposure along with its associative effects on aging processes.

**Methods:**

We imported the results of the genetics-based study that was a secondary analysis of five publicly available datasets (noted as Data1). The overlap of these genes with new data involving methylation data from two datasets (noted as Data2) following similar secondary analysis procedures is the basis of this study. We performed the standard statistical analysis on these datasets along with supervised and unsupervised learning to create preranked gene lists used for functional analysis in Ingenuity Pathway Analysis (IPA).

**Results:**

There were 664 genes of interest from Data1 and 577 genes from Data2. There were 40 statistically significant methylation probes within 500 base pairs of the gene's transcription start site and 10 probes within 100 base pairs, which are discussed in depth. IPA yielded 21 significant pathways involving metabolism, cellular development, cell death, and diseases. Compared to gold standards for gestational age, we observed relatively low error and standard deviation using newly identified biomarkers.

**Conclusion:**

We have identified 17 methylated genes that exhibited particular interest and potential in future studies. This study suggests that there are common trends in oxidative stress, cell development, and metabolism that indicate an association between aging processes and the effects of ionizing radiation exposure.

## 1 Introduction

Biological aging is an inevitable process characterized by the progressive decline of cellular and physiological functions, leading to increased vulnerability to age-related diseases and mortality. This complex phenomenon results from dynamic interactions between genetic, epigenetic, and environmental factors. In recent years, there has been an interest in deciphering the molecular mechanisms that underlie accelerated aging induced by exposure to ionizing radiation (1–3) Ionizing radiation, encompassing X-rays and gamma rays, carries sufficient energy to ionize atoms and molecules, generating highly reactive free radicals and causing cellular damage. The biological repercussions of ionizing radiation are extensively studied due to its pervasive use in medical diagnostics and radiation therapy, as well as the potential risk of exposure during nuclear accidents or space exploration. As such, a central mechanism through which ionizing radiation expedites biological aging is by inducing DNA damage (4). Radiation-induced double-strand breaks (DSBs) and oxidative DNA damage trigger intricate repair processes, leading to the accrual of genetic mutations and genomic instability. These genetic alterations are closely associated with cellular senescence, a hallmark of aging, and are implicated in the elevated risk of cancer and other age-related disorders (5). A review article thoroughly explores the dependent association between radiation damage and biological aging theory, leveraging modern technology to revisit this significantly complex topic (6).

Understanding the influence of ionizing radiation on biological aging necessitates consideration of the hallmarks of aging, an ensemble of interconnected cellular and molecular processes that collectively contribute to aging (7). These hallmarks include genomic instability, telomere attrition, epigenetic alterations, proteostasis loss, cellular senescence, mitochondrial dysfunction, and altered intercellular communication. Epigenetic modifications have recently gained substantial attention for their pivotal roles in orchestrating these hallmarks (8). Estimating biological age, in contrast to chronological age, constitutes a crucial strategy for evaluating the consequences of ionizing radiation on aging processes. Biological age offers a more precise representation of an individual's physiological state and susceptibility to age-related diseases. Gold standard methods for estimating biological age rely on DNA methylation-based clocks, such as the Horvath and Hannum clocks, which leverage epigenetic modifications as reliable aging biomarkers (9, 10). These clocks have been validated across diverse tissues and cell types, demonstrating their ability to predict health outcomes and mortality rates.

Computational biology has become an indispensable tool for unraveling the relationship between ionizing radiation, epigenetic modifications, and biological aging. The secondary analysis of existing multiomics datasets has proven to be a valid approach for investigating the epigenetic effects of radiation on aging (11, 12). These datasets typically encompass genetic, epigenetic, and transcriptomic profiles of individuals exposed to radiation, providing a rich resource for in-depth exploration. The secondary analysis has allowed researchers to harness existing resources and uncover novel insights by applying advanced data science and bioinformatics techniques (13). It can facilitate the identification of radiation-responsive biomarkers, epigenetic modifications, and gene expression changes associated with accelerated aging (14). Moreover, it can enable the construction of gene regulatory networks, shedding light on the regulatory mechanisms that underlie these effects. No matter the purpose or goals, researchers can access several valuable resources for data acquisition. Large-scale collaborative initiatives, such as The Cancer Genome Atlas (TCGA) and the Gene Expression Omnibus (GEO), offer comprehensive multiomics datasets, encompassing DNA methylation, gene expression, and clinical information for diverse cohorts (15, 16). These resources empower researchers to explore the epigenetic and genetic landscape of radiation-exposed individuals and their aging phenotypes.

Epigenetic alterations, including DNA methylation changes, histone modifications, and non-coding RNA expression, have emerged as central players in mediating the effects of ionizing radiation on biological aging. Radiation-induced epigenetic modifications can directly influence the expression of genes involved in DNA repair, cellular senescence, and oxidative stress responses (17–19). These epigenetic changes may contribute to the aging phenotype observed in radiation-exposed individuals. Moreover, radiation-induced epigenetic modifications have been implicated in the regulation of telomere length, another hallmark of aging (20). Telomeres, protective caps at the ends of chromosomes, shorten with each cell division, and their length is a crucial determinant of cellular senescence. Radiation-induced changes in DNA methylation and histone modifications can influence telomere maintenance, potentially accelerating the aging process.

The epigenetic association between ionizing radiation and biological aging represents an opportunity to revisit with modern computational methods and multiomic techniques. By harnessing the power of existing datasets and advanced analytical tools, researchers can uncover the intricate mechanisms that drive radiation-induced aging, providing valuable insights for both biomedical engineering and personalized medicine. This research not only contributes to our fundamental understanding of aging processes but also holds the promise of identifying potential therapeutic interventions to mitigate the adverse effects of radiation exposure on human health. In this study, we use the findings from a genomic-only study on a radiation-age association to explore the epigenetic landscape of the effects of ionizing radiation. We specifically examine high-dose (2Gy) exposure to identify differentiating probes and understand the underlying methylation and gene expression changes. This potentially identifies genes of interest to target as control mechanisms in studying radiosensitivity or biological age.

## 2 Materials and methods

### 2.1 Data characteristics

**Figure 1:**
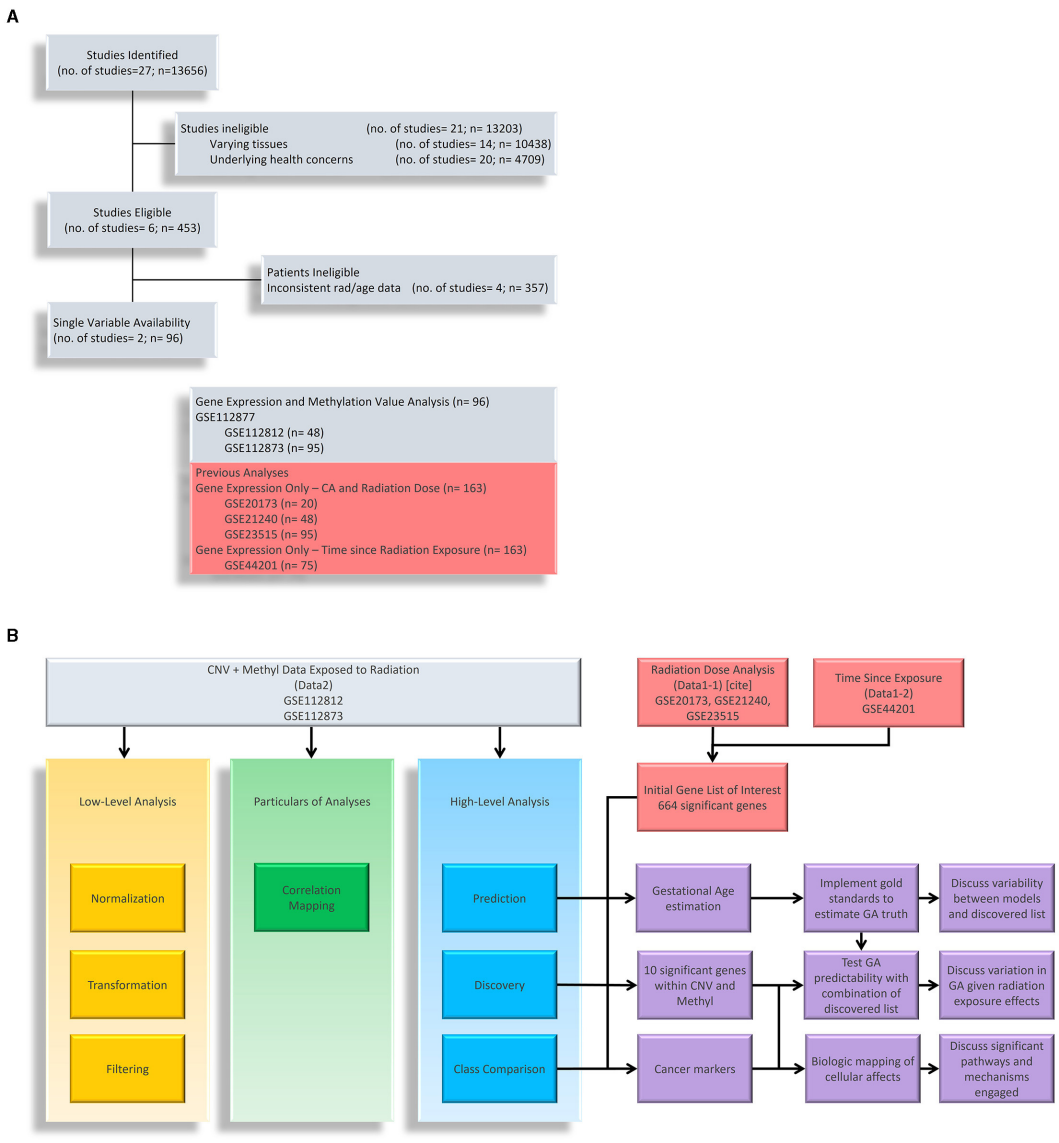
Research Flow Chart. **(A)** Shows down selection of potential datasets with methylation data on cells exposed to ionizing radiation. We have listed datasets from previous studies that are used in conjunction with gene expression. **(B)** Shows the research flow of this study. We have used methylation to understand the layered epigenetic landscape of radiation exposure from low-level analysis, particulars of analyses, and high-level analysis. Gene expression data (noted as Data1-1 and Data1-2) are used to identify genes of interest in which we take the intersection with methylation data to explore aging processes, significantly methylated genes, and disease markers.

The epigenetic aspect serves as a continuation of a multiomic exploration into the biological effects of radiation exposure. The genetics foundation that we carry over is noted as Data1, which is shown in red in Figure 1. Specifically, Data1-1 (*n* = 91) include dose analysis with sex and age as clinical factors while Data1-2 (*n* = 75) includes sex as a secondary clinical factor (21). GSE21240, GSE23515 (22), and GSE20173 (23) (collectively referred to as Data1-1) included a total of 163 originally collected samples (of which 91 are utilized in this analysis). The samples were obtained with a chronological age ranging from 21 to 64 years, who were exposed to various levels of ionizing radiation, including 0 Gy (controls), 0.1Gy, 0.5Gy, and 2Gy. GSE21240 was a study of peripheral blood mononuclear cells (PBMCs) collected from 6 individuals, following 2 different blood preservation methods, performing RNA extraction immediately or 3 h after an ex vivo exposure to 0.5 Gy of Cesium-137 gamma rays for one minute. In total, 48 samples were analyzed GSE23515 studied peripheral blood cells from 24 different donors (95 samples in total, as one sample was lost) exposed ex vivo to 0 Gy (controls), 0.1 Gy, 0.5 Gy, and 2 Gy at a dose rate of 0.82Gy/min from Cesium-137 gamma radiation to study radioactive responses between sex and smoking behavior. GSE20173 analyzed the miRNA expression profile of peripheral blood lymphocytes incubated for 4 and 24 h in normal gravity (1g) and in modeled microgravity after irradiation with 0.2 and 2 Gy of gamma rays (5 participants across 4 conditions). In GSE44201 study, we then performed time-dependent analysis on another dataset that focused on genetic effects of radiation sometime after exposure (noted as Data1-2; *n* = 75) (24). The original study was an analysis of human peripheral blood, collected 5 healthy donors, that was exposed to varying levels of gamma-ray radiation and evaluated up to 48 h after exposure. For the overlap with our data studies, we only focused on time since exposed to 2 Gy. We also identify the significant gene changes with respect to sex and age. This genetics-only analysis of Data1-1/2 was used to set a foundation and identified an intersection with significantly methylated genes in this portion.

At its core, this study aimed to explore epigenetic changes in samples exposed to radiation levels that overlap with our previous study, and we primarily explored Gene Expression Omnibus (GEO) and NASA's GeneLab repositories for datasets. Figure 1 shows a breakdown of potential studies for secondary analysis (light grey) and how we utilize chosen sets (shown in yellow, green, blue, and purple boxes for respective purposes). In Figure 1A, we focused on identifying datasets with methylation data and initially found 13,656 samples. Narrowing down on clinical factors and holding variables constant, we removed those with varying tissues (brain, skin, lung, etc.), underlying health concerns that may bias expression values (cancer), and those that did not overlap with the radiation levels of the previous study (the only common dose level became 2Gy). We ended with two studies that belonged to a parent dataset, meaning that two strains aimed toward the same goal: GSE112812 (Strain 1; *n* = 48) and GSE112873 (Strain 2; n=48) falling under GSE112877 (25). This study analyzed delayed genetic and epigenetic radiation effects that may trigger radiation-induced carcinogenesis. The researchers explored cloned descendants of fetal fibroblasts irradiated to a single dose of 2 Gy of X-ray and observed copy number variation (CNV) and methylation changes in genes. The combined dataset is referred to as Data2 (n=96) in which we downloaded the raw data of both strains separately so that they could be processed to remove the batch effect of strains. The demographics of these datasets are summarized in Table 1. Again, our focus is on the samples exposed to 0 or 2 Gy to associate with age with interest in clinical factors such as sex. The gestational age of Data2 was determined using R's methylClock v1.2.1 package with further elaboration provided later for conducting our estimation analysis (26).

**Table 1:** Demographics of the five datasets we use to analyze the association between radiation exposure and aging processes.

		**Gene Exp. (Data1)**				**Methyl** & **CNV**		
		**Dataa1-1**			**Data1-2**	**Data2**		
		**GSE20173**	**GSE21240**	**GSE23515**	**GSE44201**	**GSE112812**	**GSE112873**	**Total**
Sex	Male	-	40	48	15	-	-	103
	Female	-	8	47	15	-	-	70
Chronological age (yrs)	Minimum	-	21	21	20	-	-	-
	Maximum	-	64	45	53	-	-	-
Biological age (wks)	Minimum	-	-	-	-	12.09	12.41	-
	Maximum	-	-	-	-	42.77	41.47	-
Radiation Dose (Gy)	Control (*D*_*T*_ = 0)	10	24	24	15	13	22	108
	High Rad (*D*_*T*_ = 2)	10	0	23	15	35	26	109
Time since exposure (hrs)	6	-	-	-	10	-	-	10
	24	-	-	-	10	-	-	10
	48	-	-	-	10	-	-	10
	Total	20	48	95	30	48	48	289

### 2.2 Recreating epigenetic workflows

With regards to Figure 1B, this subsection covers the low-level analysis (yellow box) and particulars of analysis (green box). These two, in addition to the outline of high-level analysis (blue box only) represent a recreation of methodology created particularly for epigenetic understanding (27). It is worth noting that, for data in GeneLab, fastq was the most common data form used to generate coverage files. While we did not use human data from this repository, mice data seemed promising. To explore it, methylation data were processed using nf-core/methylseq v2.3.0 (doi: 10.5281/zenodo.1343417) of the nf-core collection of workflows (28). The pipelines were executed using Nextflow v22.10.4 (29) with the below bash command. Unfortunately, several reports failed quality assurance as well as a very small number of probes relative to datasets found on GEO. Additionally, there was a lack of overlapping annotated probes to combine data from different repositories. of the nf-core collection of workflows (28). The pipelines were executed using Nextflow v22.10.4 (29) with the below bash command. Unfortunately, several reports failed quality assurance as well as a very small number of probes relative to datasets found on GEO. Additionally, there was a lack of overlapping annotated probes to combine data from different repositories.

For Data2 processing and normalization, we used R v4.2 library minfi v1.42 to turn raw idat files into usable count data (30). Recreating processing checks and techniques, we plot and evaluate a histogram of M-values, beta values, and log2 transformed gene expression with a distribution curve overlayed. This is to check reasonable density plots for each before moving forward to plotting the dataset's standard deviation and MA plots. MA plots are a visualization of log fold change (*y*−*x*) vs. average intensity ((*x*+*y*)/2) of two populations *x* and *y*. For our data, we used respective irradiated samples as *x* and non-irradiated as *y*. MA plots help observe the assumption that the majority of gene expression or methylation is insignificantly changing and should be along the zero line and can be an indication of what one can expect later in the analysis, such as how many probes are indeed at a higher fold change. Finally, for low-level analysis, we split the CNV into quantiles of low, medium, and high expression. We take the corresponding probe in methylation data to plot the histogram/density plot of the average beta value along with the distance to the transcription start site (TSS). Probes of interest are those typically within 500 bp of TSS in the promoter region. Correlation mapping involves analyzing the correlation between gene expression probes and methylation. Since we do not expect CNV to be fruitful as it is used for mutations, we calculate the correlation matrix between the methylation probes of Data2 and the corresponding gene expression probes of Data1. Here, an assumption is that significant findings are methylation probes that are heavily anti-correlated with their respective gene expression probes. Bringing together these recent observations, we find that observations of extreme interest are those that have high, inverse relationship in correlation, within 500 bp of the TSS, along with significant *p*-values/log FC between irradiated and non-irradiated populations.

### 2.3 Statistical analysis

Finally, the high-level analysis emerges, which quickly becomes complex and is further elaborated upon in Figure 1B. The methylated data, represented by the blue box, is integrated with the genetics-only radiation analysis (red) to become the purple portion of the flow chart and is the bulk of our results and discussion section.

To begin, we find the overlap of the genes of interest from Data1 with those in Data2 with regards to radiation-only analysis, age-only analysis, rad-age interaction, and sex-only analysis. We aim to determine the significant genes categorized under each analysis type. Additionally, we seek to provide a holistic comparison across all categories. Since we are exploring the epigenetic landscape of radiation effects on aging, we specifically analyze a PCA plot of radiation groups with gene expression and methylation data to observe the overlap or differentiation between control and high radiation-exposed groups. Along with just the number of overlapping genes, we use the Circos v0.4.10 library in R to create a circular plot for an initial understanding of expression levels, clustering, and trends when analyzing expression and methylation (31). We perform Student t-tests on Data2 to create volcano plots to best visualize p-value and log2 fold change significance. We then look at the overlap between significantly changing M-values and gene expression values to evaluate potential control mechanisms. We create another circos plot and heatmap to visualize values and trends.

### 2.4 Functional and clinical relevance

We used k-means clustering and identified 5 groups of interest (also shown on the circos plot and discussed later). We then create preranked gene lists for enrichment analysis divided into these 5 groups. Data are then analyzed using IPA (QIAGEN Inc., https://digitalinsights.qiagen.com/IPA) (32). We used both p-values and log2 fold change between radiation groups to run Individual Pathway Analysis (IPA) to flag significantly changing and linked biological pathways and diseases associated with our findings. As previously mentioned, we use methyClock v1.2.1 to evaluate identified markers with aging (26). Since Data2 are cloned fetal cells, we implement methylated gestational age (GA) estimation techniques found in this library listed as Bohlin, Epic, Knight, Mayne, Lee CPC, Lee reference RPC, and Lee RPC. Using all available probes of Data2, we get the estimated GA from these techniques to serve as our reference or “truth” value. Notably, we recognize that these are the estimations and, truth is relative. This is a limiting assumption as we develop a rad-age association. We then use our identified, significant rad-age methylated probes to predict sample GA and analyze the error rates compared to these established techniques. We plot the errors in weeks using root mean squared (RMS) error and discuss variation.

## 3 Results

### 3.1 Understanding the epigenetic landscape

Our analysis begins with understanding the epigenetic landscape of the data we are using. With specifics outlined in the methodology section, we present results covered in the “low-level analysis” and “particulars of analysis” portions represented by yellow and green boxes, respectively, of Figure 1 where we recreate epigenetic workflows to be implemented for our purposes (27). Overall, this is to gain a detailed understanding of the data and how it pertains to the problem at hand.

We use the R minfi library to process the raw idat files from GEO. We used the preprocessFunnorm function on the red-green channel set object to generate functionally normalized ratio set data. Funnorm extends the idea of quantile normalization from the same library, and we use getBeta, getM, and getCN functions to extract beta-values, M-values, and copy number variant data, respectively. Similar to the study we replicated, we show a histogram and density plot of the data to check for expected shape and intensity shown in Supplementary Figure S1. Since we are focusing on epigenetic and genetic relations, we do not use the CNV data for downstream analysis. We show standard deviation blots of beta-values and gene expression along with MA plots for M-values and gene expression in Supplementary Figure S2 where we see expected results of the majority of probes having insignificant fold change. Finally, in Supplementary Figure S3, we again mirror our reference on exploring beta values for different quantiles of copy number variations and the corresponding distance from the gene's transcription start site (TSS). Our primary observation centers around the significant decrease in hypermethylated genes with the near constant scatter of expected distance from the TSS.

### 3.2 Statistical and high-level analysis

**Figure 2:**
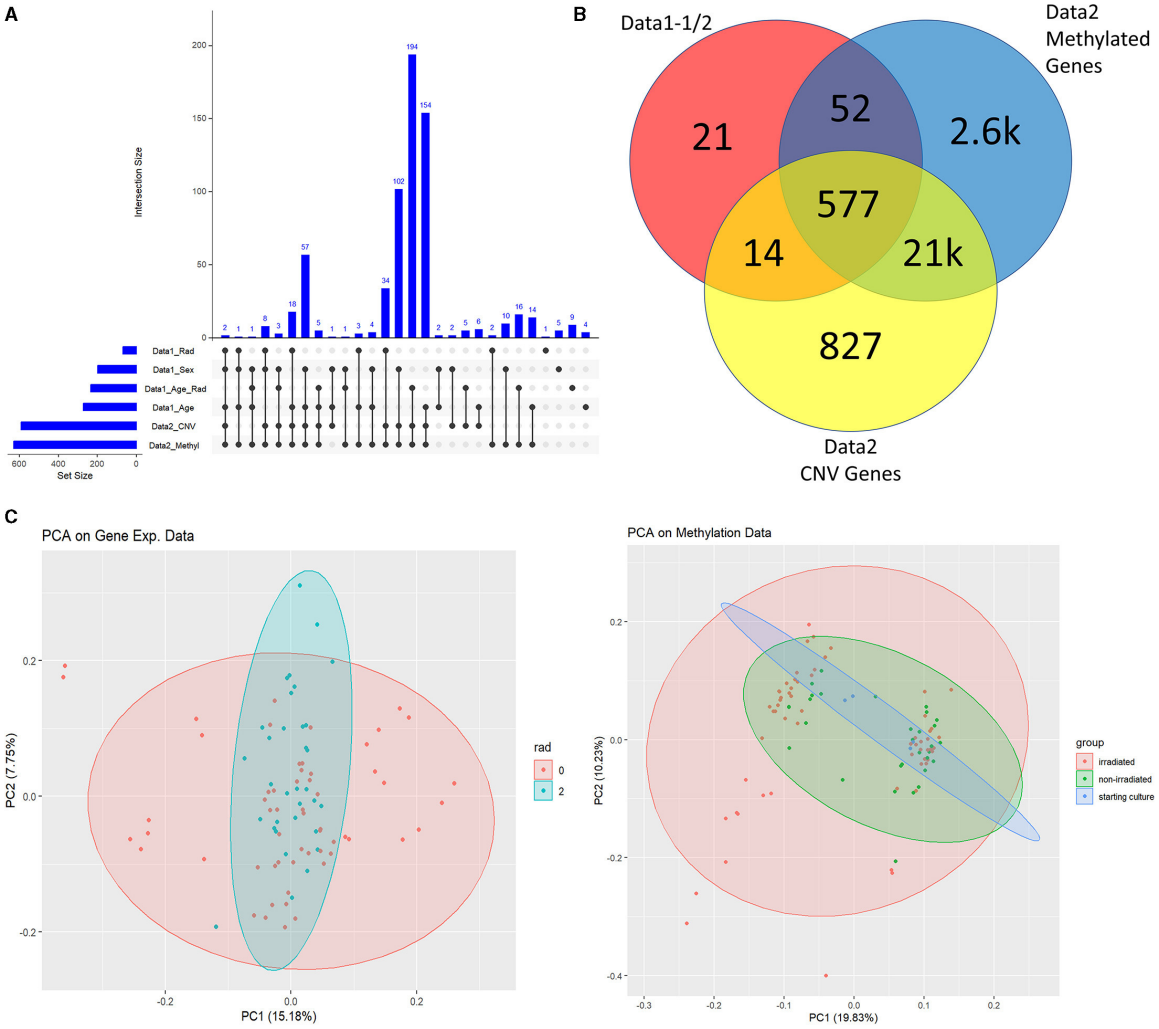
Data1 and Data2 overlap. **(A)** Is the overlap of significant genes shared between Data1 and Data2 for listed subcategories. We identified significantly changing genes with respect to sex, age only, radiation only, and the interaction between radiation and age. These 664 genes from various clinical factors are the ones shown in the red circle of **(B)**. **(B)** shows the overlap between Data1 significant genes with the statistically significant probes of Data2 with respect to methylation and CNV. **(C)** are PCA plots of gene expressions of Data1 and M-values of Data2 with ellipsoids around group of interest such as irradiated (2Gy) and non-irradiated (0Gy). Since we focus on linking methylation to expression, we continue this study with the methylation data of Data2 and the gene expression data of Data1 (instead of the CNV data of Data2).

Understandably, most of our findings deal with the high-level analysis of epigenetic data indicated by the blue and purple sections of Figure 1. We started by importing all t-test results from Data1-1 and Data1-2 (collectively referred to as Data1) where we extracted genes that were significantly different between populations of sex, age, radiation exposure (only 0 Gy vs. 2 Gy), and the interaction test between the same radiation exposure levels with age (referred to using the R notation of rad*age or in plots with the underscore in place of the asterisk). We examined the overlap between this compiled list of 664 significant genes of interest from Data1 and those present in Data2. This alternate form of a Venn diagram using the R package UpSetR is shown in Figure 2A where vertically connected dots represent the overlap in the label category. This shows the breakdown of overlap by the t-test while a summarized gene list is shown in Figure 2B where we see 577 of the 664 genes are also in Data2. As mentioned, since CNV data are not necessarily relevant toward understanding the epigenetic landscape or control mechanisms we continue with the overlap between gene expression levels of Data1 with corresponding methylated levels in Data2 to compare trends. This analysis leads to Figure 2C where we see unsupervised clustering via principal component analysis (PCA) of irradiated groups of gene expression and methylation data, revealing a broad overlap between the two groups. A list of all genes and their overlapping groups (used to created Figure 2A) are presented in Supplementary Table 1.

**Figure 3:**
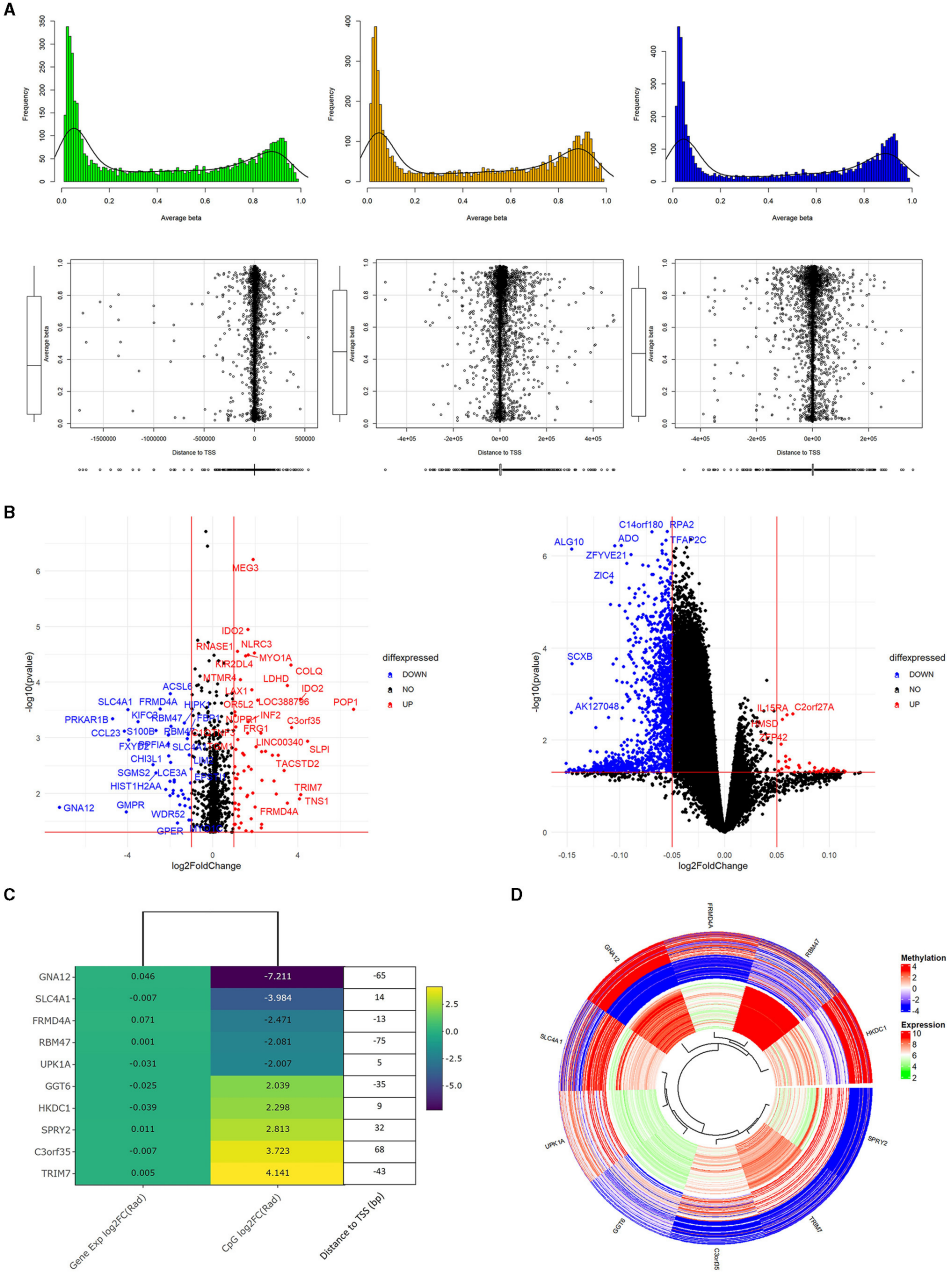
Methylation analysis supplementing genetic findings. **(A)** is a plot of the processed Data2 sites of interest from Data (previous figure). We separate Data2 into three quantiles of gene expression to view average beta trends (top) as well as distance of the methylation probes to the corresponding gene's transcription start site (bottom). Of note is the general similar shape in average beta distribution and there iss increased variance in TSS distance with increased gene expression. **(B)** are volcano plots of *p*-value vs. log2FC of radiation only analysis between methylation (left) values and gene expression (right). These are only the probes that correspond to genes of interest from the original 664 list. The methylation values here are those with *p* < 0.05, –log2FC(radiation)–>2. Additionally, 40 of these probes also had a –TSS distance– < 500bp and 10 probes with a distance < 100bp. Looking specifically at these 10 probes are the focus of **(C, D)**. **(C)** shows a heatmap of the 10 methylation probes with the corresponding gene expression colored to log2FC(radiation) of the respective dataset. Added onto the right of the heatmap is the basepair distance to the TSS from the annotation file for the methylation probe. **(D)** is methylation (out ring) and gene expression (inner ring) of genes from the heatmap. Specifically, the study highlights the varying methylation rates while somewhat consistent gene expression.

We conducted Student's t-test on all Data1 probes which included those found in Data2 to potentially identify overlapping genetic/epigenetic markers to eventually control. Figure 3A shows a familiar plot structure by showing a beta histogram for different quantiles of gene expression and the corresponding distance from the gene's transcription start site. With less probes of interest, we are able to see a bit more resolution trying to see trends. Of note with beta values, we identify an increase in gene expression corresponded to a lower percentage of methylated genes as somewhat indicated with the hypermethylated hump pulling left as we go across the plots. Meanwhile, the scatterplots of methylation probe TSS distance seemed to increase in variability with increased gene expression. Figure 3B represents those results with volcano plots of the Data1 gene expression and Data2 methylation data independently evaluated. There were no genes of significant fold change, so we picked an artificial threshold for plotting. Of the methylation probes that had a p-value < 0.05 and log2FC(radiation)>2, 40 probes were within 500 base pairs of the gene's transcription start site and 10 within 100 base pairs. While we performed the analysis on both gene expression and methylation data for completion, we again proceed with evaluating methylated genes. Figures 3C, D show the methylation results, plotted with corresponding gene expression values from Data1. Figure 3C is a fold change heatmap showing the aforementioned 10 methylation probes matched to the closest gene symbol along with the gene expression log2FC. Next to the colored fold-change values is a list detailing the distance to the TSS for that methylated gene. Figure 3D shows those same 10 probes in a circos plot with the methylation values on the outer ring and corresponding gene expression (regardless of significance) on the inner ring. Here, it is easier to see the relationship between gene and epigenetic changes. Raw t-test findings (to include the change and *p*-values that were plotted here) are presented in Supplementary Table 2.

**Figure 4:**
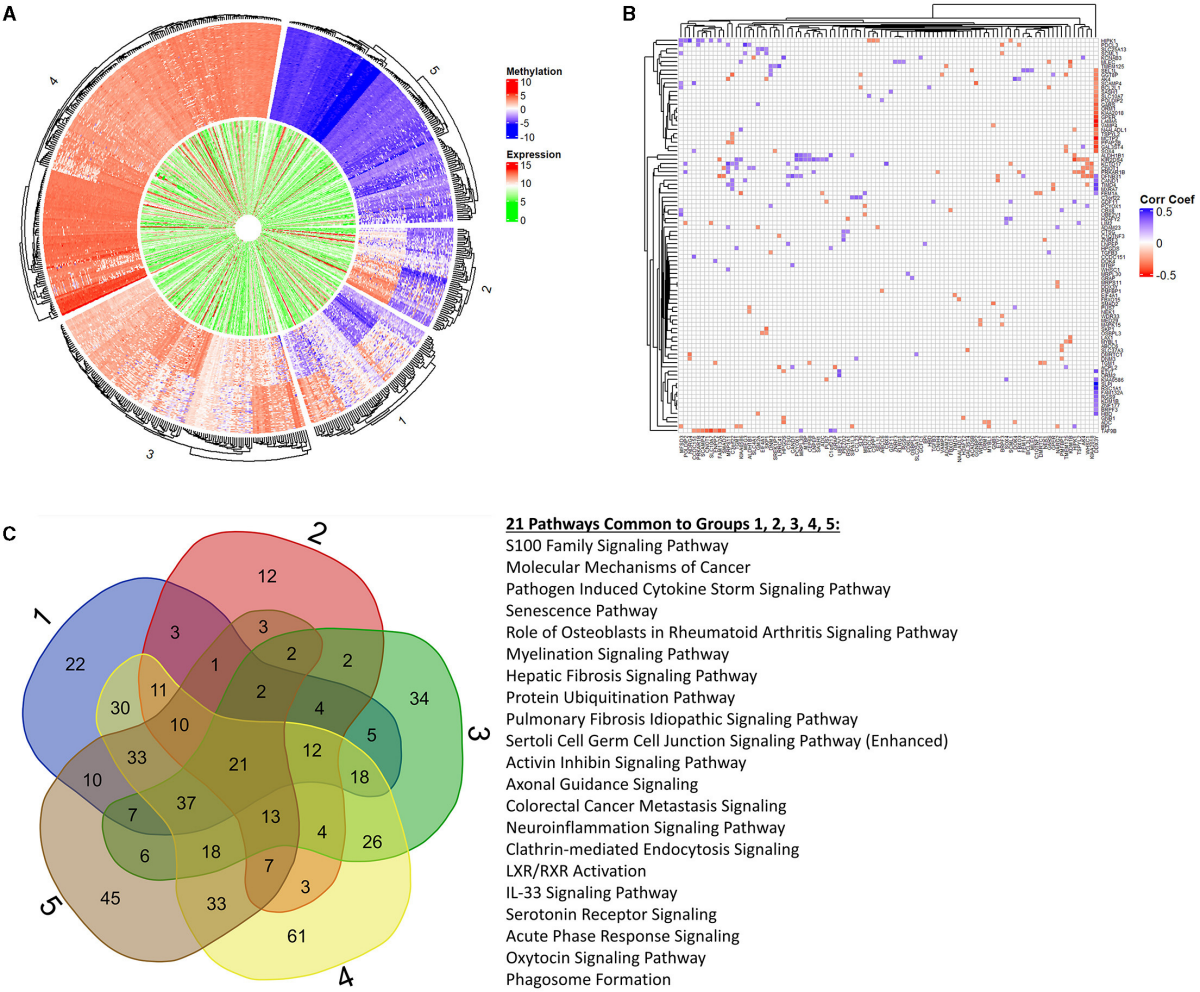
K-means clustering, correlation, and pathway overlap. This figure represents a more holistic analysis of the 664 genes of interest in relation to methylation values. First, **(A)** is a circos plot of methylation values (outer ring) and corresponding gene expression (inner ring). We performed k-means clustering to identify five distinct groups, which are represented by this plot that is split into labeled sections on the outside. We created preranked lists of all groups based on the p-values and log2FC(radiation) to feed into Ingenuity Pathway Analysis (IPA) for functional analysis. Looking at all probes regardless of the group, we calculated a correlation matrix between methylation probes (columns) and gene expression (rows). To better visualize significant changes in groups 1 and 2 due to changing methylation trends (which may or may not align with the changes from irradiation), we extracted the top 1% of correlation values to plot in **(B)**. We see complex biology at play with changes in a methylated gene both positively and negatively correlated to the changing expression in other genes, and vice versa. **(C)** represents the overlapping pathways from IPA between each clustered group. Next to the Venn diagram are the 21 pathways common among all groups. We see a trend in immune response, cell death, age-associated diseases, and cancers through these pathways as well as in the extended list provided in the Supplementary material referenced in the manuscript.

Next, we focused on overlaying methylation levels with gene expression without digging into the genes themselves. While the previous paragraph/figure explores all significant probes of Data2 corresponding to their gene expression, this section starts with the significant gene list of Data1 (664 genes of interest mentioned) and explores the available methylation values. We used k-means clustering of methylation values to identify five groups of similar expressions. Figure 4A shows a circos plot of these clusters along with corresponding gene expression. The outer ring, representing the methylation values that are ordered by clusters, we see one group of hypomethylation, one group of hypermethylation, one of seemingly unmethylated, and two where there is some type of changing inverse relationship. The inner ring, as mentioned, is the gene expression of the corresponding probe to the plotted methylation values. While there might seem some correlation, it is not ordered and much harder to tell with the naked eye. While we dig into specifics more, this is merely a visualization step to see that there is indeed some trend to be explored. Since we are interested in the understanding landscape of radiation effects, Table 2 is a summary of these five clusters when looking at irradiated vs. non-irradiated samples. Due to the changing relationship, the probes of clusters 1 and 2 are pulled for correlation plotting. Specifically looking for positive or negative correlation between methylated genes and expression, Figure 4B shows the top 1% of most absolute correlated values. For example, upon reviewing this plot, as will be discussed in the next section, the methylation of *DDX3Y* (far right column) demonstrates a significant positive-correlation (blue) with the expression of 15 genes, such as *HBD, BRPF3, ZNF177, KCM1B, RGS9, FAM132A, RSC1A1, SLPI, KIAA0586, ETV7, FEM1A, MXRA7, TIMD4, CAND1*, and *DFNB31* while anti-correlated (red) with the expression of 20 genes, such as *SEL1L1, GGT8P, AK4, SCAMP4, BCL2L1, SASH1, SLC10A7, POLDIP2, GARS, ORM1, KIAA2018, GPER, LAMA5, VAMP4, NAALADL1, TSPYL2, MCTP2, PPAP2B, GAL3ST4*, and *SOX4*. The raw correlation values can be found in Supplementary Table 3.

**Table 2:** Sample split by k-means cluster group on hyper- or hypo-methylated probes.

**Cluster group**	**Irradiated**		**Non-irradiated**	
	**Hypomethylated**	**Hypermethylated**	**Hypomethylated**	**Hypermethylated**
1	28	33	22	9
2	35	26	9	22
3	0	61	0	31
4	0	61	0	31
5	61	0	31	0

### 3.3 Functional and clinical relevance

Overlapping statistical findings with functional analysis, we create preranked lists based on p-values and log2FC(radiation) from the five k-means clusters of the original genes (611 found within these data of the 664 genes of interest). These preranked lists were analyzed using Ingenuity Pathway Analysis (IPA) for identifying significant pathways and diseases. Figure 4C shows a Venn diagram of output pathways with those intersecting 21 among all groups specified. Of particular note are the senescence, myelination signaling, neuroinflammation, and IL-33 signaling pathways overlapping in all groups, which clearly ties to aging processes. Seeing such pathways that would be tied to old age in fetal cells supplements a rad-age association with the epigenetic background. Looking specifically at cluster groups 1 and 2 (inverse relationship of M-values), we see pathways involved in the metabolism (*PFKFB4* signaling and fatty acid α-oxidation), cellular development (Hippo signaling and putrescine biosynthesis/degradation), cell death (MYC mediated apoptosis and induction of apoptosis by HIV1), and disease (*THOP1* in Alzheimer's disease, Huntington's disease, and colorectal cancer metastasis) to name a few. These intersecting pathways, and others of significant value to a rad-age association, are discussed more in the next section. The labeled k-means groups for each probe is listed in Supplementary Table 4 while all IPA results for these groups (molecules, pathways, overlapping groups, etc.) are presented in Supplementary Table 5 and 6.

**Figure 5:**
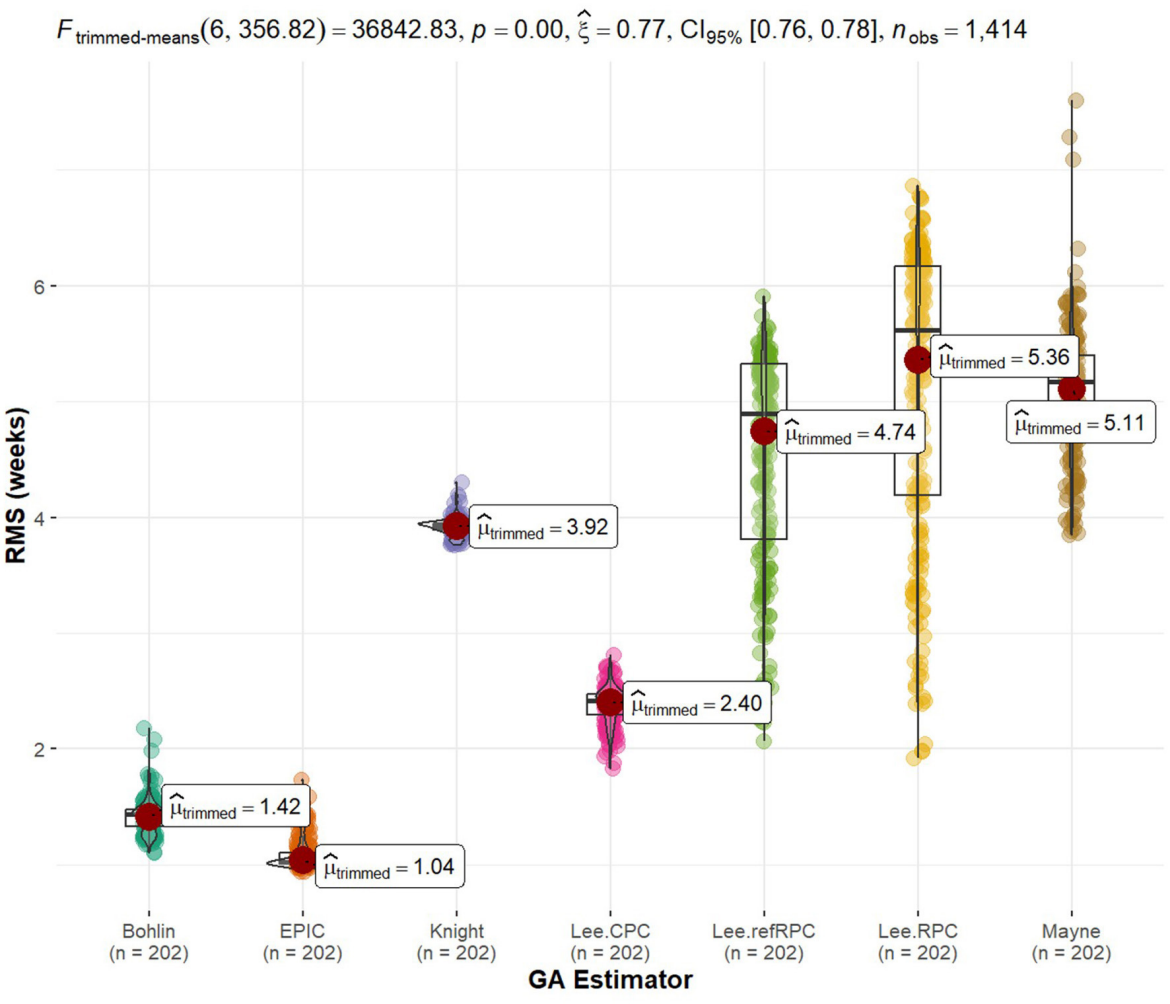
Rad-age predictability error of gestational age. We implemented gold standards of biological age estimators, such as gestational age (GA) for fetal cells given in weeks. The GA is held as truth or error = $\hat{\mu }$ = 0. We then created generalized linear models to evaluate the GA predictability of the 664 genes of interest. The error of these linear models was calculated as the root mean squared (RMS) error in reference to the estimated GA. Since truth is relative or we may doubt the accuracy of estimation techniques, we are more interested in the relative error of our models as opposed to the estimated age itself. The spread of error is plotted for each GA technique. It is noteworthy that none of the 664 genes of interest from our rad-age association overlap with a gene used by the gold standards of GA estimation. Additionally, tight groupings (low variance) of error are observed between our linear models and most of the GA techniques.

Tying back to the radioactive effects on biological aging, we implemented standard gestational age (GA) estimators in R as our reference value so we could explore the predictability of our statistically significant methylation probes. Using a 60/40 split between training and testing data, we fit a generalized linear model to the data and calculated the RMS error to each of the GA references. The GA of each sample in itself was not of interest to us because truth is relative and there is currently no way to tell how close to truth they are. Instead, we are interested in how close these methylated genes were to the standards. Figure 5 shows boxplots of error with models listed along the x-axis and RMS error to the standards along the y-axis (33). An interesting note is that none of these statistically significant probes when comparing radiation were used to implement the gold standards. We were truly using new potential markers to estimate age. Additionally, it is interesting to see tight groupings (little variation in error) on non-Lee models and there is a low relative error in these models. The train/test split of samples can be found in Supplementary Table 7 while the statistically significant probe list was previously mentioned in Supplementary Table 8.

## 4 Discussion

Our first thing of note is the difference in the quantile separation of beta values and TSS plots in Supplementary Figure S3 and Figure 3A compared to the findings of Singhal et al. (27). In our data, we have relatively unchanged histogram shape with increasing gene expression in dealing with radiation exposure with spikes in both hypo-and hyper-methylated regions. However, Singhal et al. had primarily hypomethylated genes in medium and high gene expression with almost a noise-like histogram of beta values among low expressed genes. This seems to be our first characterization of expectations when dealing with the epigenetic landscape of those exposed to ionizing radiation. With regards to significant, low absolute distance from the TSS of v, given our focus on gene expression instead of mutation rates, we look at the regions of [-500, 500] and [-100, 100]. In the highly expressed genes, 35.8% (1895 of 5289) and 13.3% (704 of 5,289) of the methylation probes were in these regions. There were 33.8% and 12.1% (out of 5223 probes) in these regions among medium gene expression. And lastly, 35.7% and 12.3% (out of 4,810 probes) in these regions respectively amongst low gene expression. Additionally, since we brought up a potential shift in hypermethylation, we looked at the frequency of beta value counts in that region of the histogram. High gene expression saw a peak count of 147 at 93% methylation, medium gene expression peaks at 124 counts each of 92–93% methylation, and low gene expression peaks at 95 counts each of 92-93% methylation. This can help research in recognizing this epigenetic landscape with a trend of increased hypermethylation of genes with increased gene expression when exposed to ionizing radiation while maintaining a ratio of distances to the gene's transcription start site.

Looking at the 10 genes highlighted from Figures 3B, C, the first to draw attention is Gene Protein Subunit Alpha 12 (*GNA12*) which is predicted to enable the GTPase activity and involved in regulating TOR signaling. This is arguably the more significant finding because the GTPase activity with another gene has been shown to promote radioresistence in cells, and TOR signaling is directly linked to aging processes (34–37). The Solute Carrier Family 4 Member 1 (*SLC4A1*) gene encodes a protein part of the anion exchanger in the red blood cell plasma member where it is involved in CO_2_ transport from tissues to lungs. This becomes an interesting finding in the perspective of radiation being a known causal factor of cancers in high proliferating cells (such as in blood forming organs) as well as CO_2_ (38, 39). FERM Domain Containing 4A (*FRMD4A*) encodes a protein that regulates epithelial cell polarity. While associated with nicotine dependence, this has more relevance with a rad-age association because of its connection with Alzheimer's disease (40). RNA Binding Motif Protein 47 (*RBM47*) enables RNA binding activity and is predicted to act upstream of cytidine to uridine editing as well as upstream of hematopoietic progenitor cell differentiation. Since C-to-U editing often involves the hydrolytic breakdown of amino acids for energy following excess protein intake, this is yet another gene found to be tied to energy in some regard (41). Additionally, a repeat of some sense is seeing reference again to blood cells. With observed changes from radiation exposure on a gene involved in hematopoietic cell differentiation, tied to one of the hallmarks of aging is stem cell exhaustion, RBM47 is creating a loose corroboration to a rad-age association (42). Gamma-Glutamyl transferase 6 (*GGT6*) belongs to the GGT family that is a membrane-bound extracellular enzyme that is key to glutathione homeostasis as it provides synthesis substrates. This is indirectly significant with regards to a rad-age association because glutathione is a powerful antioxidant produced by the liver to deal with free radicals, peroxides, and heavy metals (43). Following connections with the liver, Hexokinase Domain Containing 1 (*HKDC1*) encodes a protein involved in glucose metabolism. Reduced expression may be associated with gestational diabetes while high expression may be associated with poor prognosis in hepatocellular carcinoma (44). C3orf35 is an alias and previous HGNC symbol for the *APRG1* tumor suppressor candidate which is an RNA gene with little known properties or associations beyond a study in 2005 that it may suppress tumor growth in breast cancer (45). The protein from Sprouty RTK Signaling Antagonist 2 (*SPRY2*) is involved in the non-cell autonomous inhibitory effect on fibroblast growth factor two signaling. It is noteworthy that a mutation in this gene has been found to inhibit MAPK pathway which is involved in tumorigenesis (46). Uroplakin 1A (*UPK1A*) codes a cell-surface protein that may play a role in normal bladder epithelial physiology and possible tumor suppression (47). Tripartite Motif (TRIM), containing 7, encodes a protein that may participate in glycogen synthesis as well as both tumor-promoting and tumor-suppressing functions in innate immunity (48). Finally, while we present all results for viewer's understanding and interpretation, we noted that it was interesting to see the methylation of *DDX3Y* both positively and negatively correlated with the expression of many distinct genes. The protein from this gene is considered to be involved in ATP binding, hydrolysis, and RNA binding (49–51). Mutations in this gene result in male infertility being on the Y chromosome. While there is connection to aging processes, the continued trend of energy molecules is an exciting find with this dataset and approach.

Shifting to IPA results while continuing the discussion of significant genes, we see five genes that are recurringly found in the most significant pathways of cluster groups 1 and 2. *BCL2L1* codes a protein in the BCL-2 family that acts as an anti- or pro-apoptotic regulator located at the outer mitochondrial membrane. More significantly to our cause, it regulates the outer mitochondrial membrane channel (VDAC) potential and thus controls the production of reactive oxygen species (52). Again, continuing a theme of energy, ATPase sarcoplasmic/endoplasmic reticulum CA2+ Transporting 3 (*ATP2A3*) encodes one of the SERCA CA2+ ATPases located in muscle cells. This enzyme catalyzes the hydrolysis of ATP and is involved in calcium sequestration associated with muscular contraction and loosely tied to radioresistence and aging separately (53, 54). G protein subunit beta 1 (*GNB1*) codes a beta subunit, which regulates alpha subunits of nucleotide-binding proteins that integrate signals between receptors and effector proteins. One such case is when beta and gamma chains are required for the GTPase activity which we saw relevant with *GNA12* (one of the 10 methylation probes within 100bp of its gene's TSS). SMAD family member 2 (*SMAD2*) encodes a protein that mediates the signal of the transforming growth factor (TGF)-beta, thereby regulating processes, such as cell proliferation, apoptosis, and differentiation, as well as associated with cardiovascular diseases - all of which give confidence in linking effects of radiation and aging (55–57). Aldehyde Dehydrogenase 1 family member B1 (*ALDH1B1*) is the second enzyme of the major oxidate pathway of alcohol metabolism. The review of the literature connects downregulation of *ALDH1B1* to numerous cancers including colorectal, pancreatic, liver, prostate, lung, brain, and breast cancers to name a few (58–64). While *ALDH1B1* may indirectly or loosely support a rad-age association, given its ties to cancer progression and radioresistence, this enzyme also perks our interest once more on being yet another metabolic constituent.

Understandably with this dataset, cellular and embryonic development pathways are highlighted as significant. We cannot know if that's due to the radiation effects on the epigenome or because of the cell types used in this study. While briefly mentioning the pathways, we can revisit knowing significant genes/molecules that play a role. Energy and metabolism was an interesting trend compared to others in that it is not directly called out in response to radiation and aging. It could be underneath mitochondrial dysfunction and deregulated nutrient-sensing for hallmarks of aging but not considered obvious. Fatty acid oxidation comes close and was highlighted in groups 2, 3, 4, and 5 primarily due to the significance of *ALDH1B1* that was just discussed. From groups 1, 3, 4, and 5 is *PFKFB4* signaling which is another significant pathway involved in metabolism. Although the gene itself was not in our gene list, we wanted to highlight its function. The protein encoded by this gene is highly expressed in cancer cells and is induced by hypoxia (65). It forces the cell to increase the amount of energy (i.e., ATP) production beyond its typical constraints (66). Overlapping with cancer pathology is the Hippo signaling pathway from groups 2 and 3 (also sharing T-Cell exhaustion signaling) which modulates the proliferation, differentiation, and survival of cells (67). Another pathway without the gene itself is *MYC* mediated apoptosis from groups 1 and 4 (along with a necroptosis and pyroptosis signaling pathway). While the gene itself plays a role in cell growth, proliferation, differentiation, and apoptosis, the pathway focuses on inducing apoptosis within a cell when survival factors are missing (68). As may be expected, a number of cancer pathways were flagged as significant from our rad-age associated gene list. Colorectal and gastrointestinal cancers were recurring while breast, ovarian, lung, prostate, and bladder cancers were also found from multiple cluster analyses.

## 5 Conclusion

In this study, we utilized genetic findings by exploring previously identified genes of interest to investigate the epigenetic landscape of ionizing radiation and its relationship to aging processes. Our primary source of results stem from conducting the secondary analysis on two publicly available datasets of fetal fibroblasts exposed to 2 Gy of radiation. The Student t-test was performed to create preranked gene lists based on the p-values and log2FC(radiation) for functional analysis in IPA where significant pathways and diseases were identified. Upon close examination of k-means clustering groups, the correlation between methylation and gene expression, and molecules that emphasized pathway results, we discussed 17 methylated genes that showed particular interest and potential in future studies: *GNA12, SLC4A1, FRMD4A, RBM47, GGT6, HKDC1, APRG1, SPRY2, UPK1A, TRIM7, DDX3Y, BCL2L1, ATP2A3, GNB1, SMAD2, ALDH1B1*, and *PFKFB4*. Many other findings (such as 40 methylation probes within 500 base pairs of their gene's transcription start site) in addition to raw pathway analysis are provided in the Supplementary material for the reader's use. We found common trends in oxidative stress, cell development/growth/death, immune response, and (in an unforeseen manner) metabolism/energy without direct links to mitochondrial dysfunction.

## Data availability statement

The original contributions presented in the study are included in the article/Supplementary material, further inquiries can be directed to the corresponding author.

## Author contributions

NR: Writing – review & editing, Software, Writing – original draft, Visualization, Methodology, Investigation, Formal analysis, Data curation, Conceptualization. SS: Formal analysis, Writing – review & editing, Software. DS: Writing – review & editing, Supervision, Project administration, Funding acquisition. SKS: Conceptualization, Formal analysis, Investigation, Project administration, Supervision, Writing – review & editing.
