# Supplementary figures and images for: Translating genetic findings to epigenetics: identifying the mechanisms associated with aging after high-radiation exposure on earth and in space
Source: Front Public Health. 2024 Mar 22;12:1333222. doi: 10.3389/fpubh.2024.1333222 (PMC10995328; doi:10.3389/fpubh.2024.1333222)

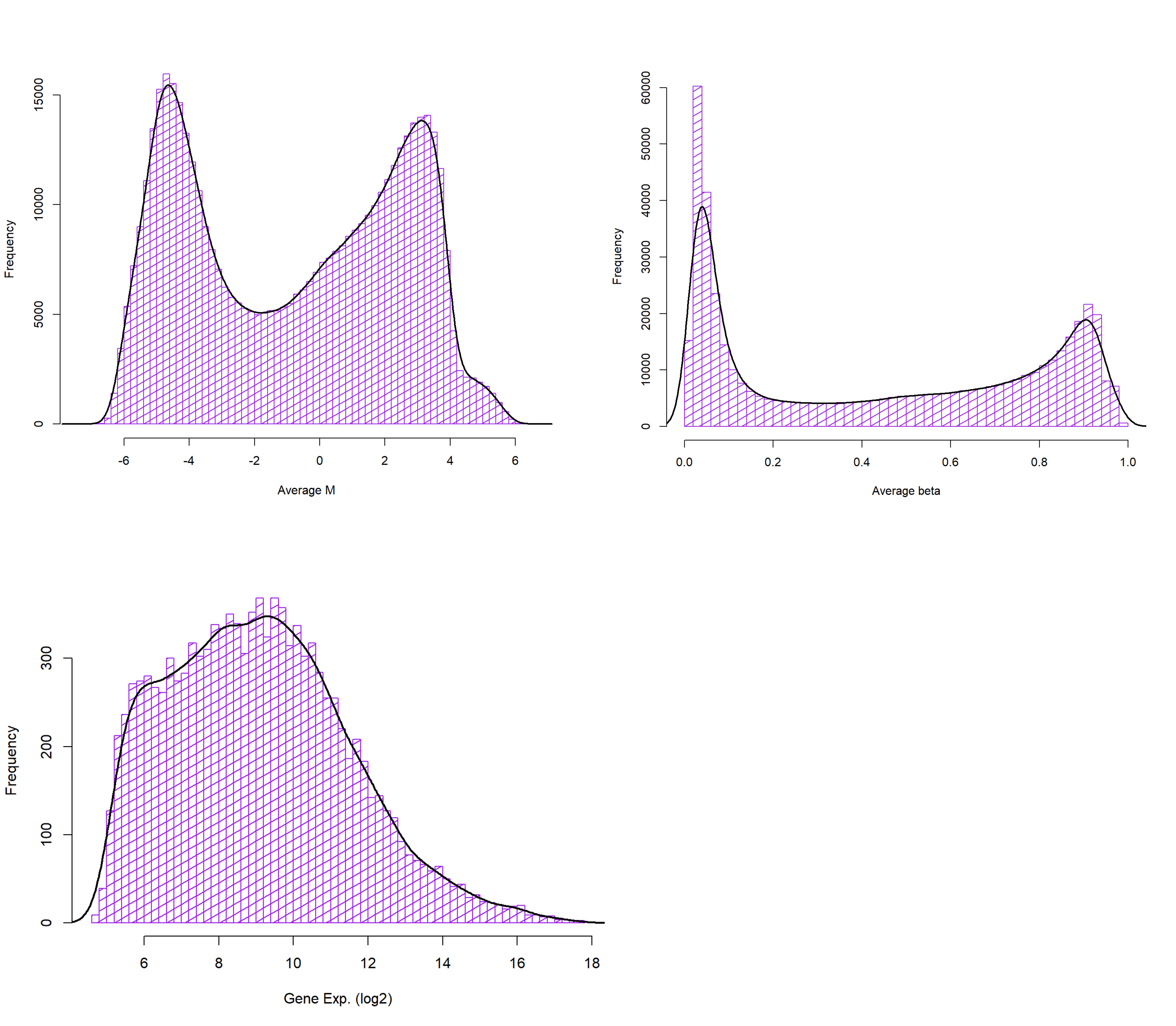

Supplement: Supplementary file 1 [file Image_1.jpg]

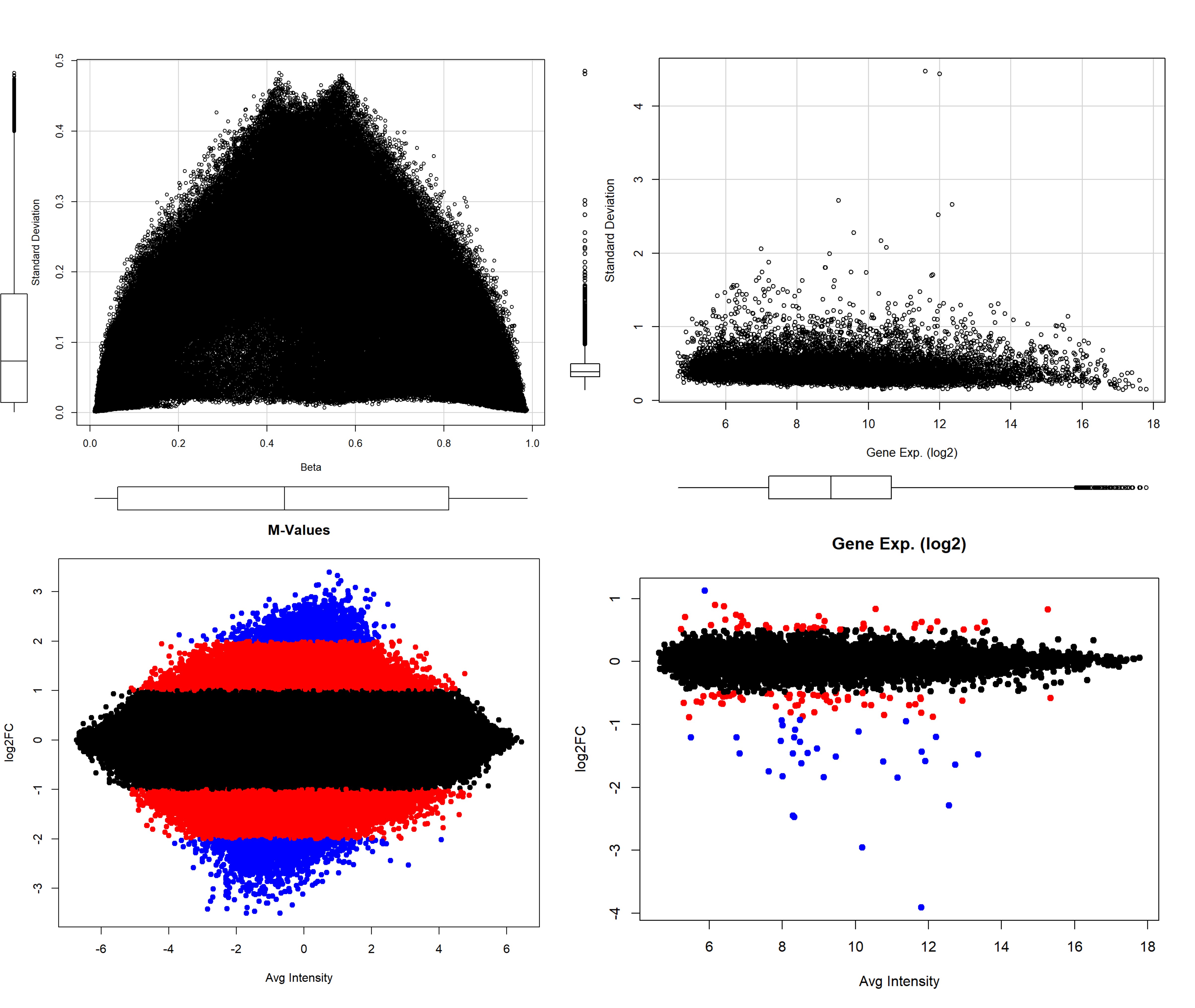

Supplement: Supplementary file 2 [file Image_2.jpg]

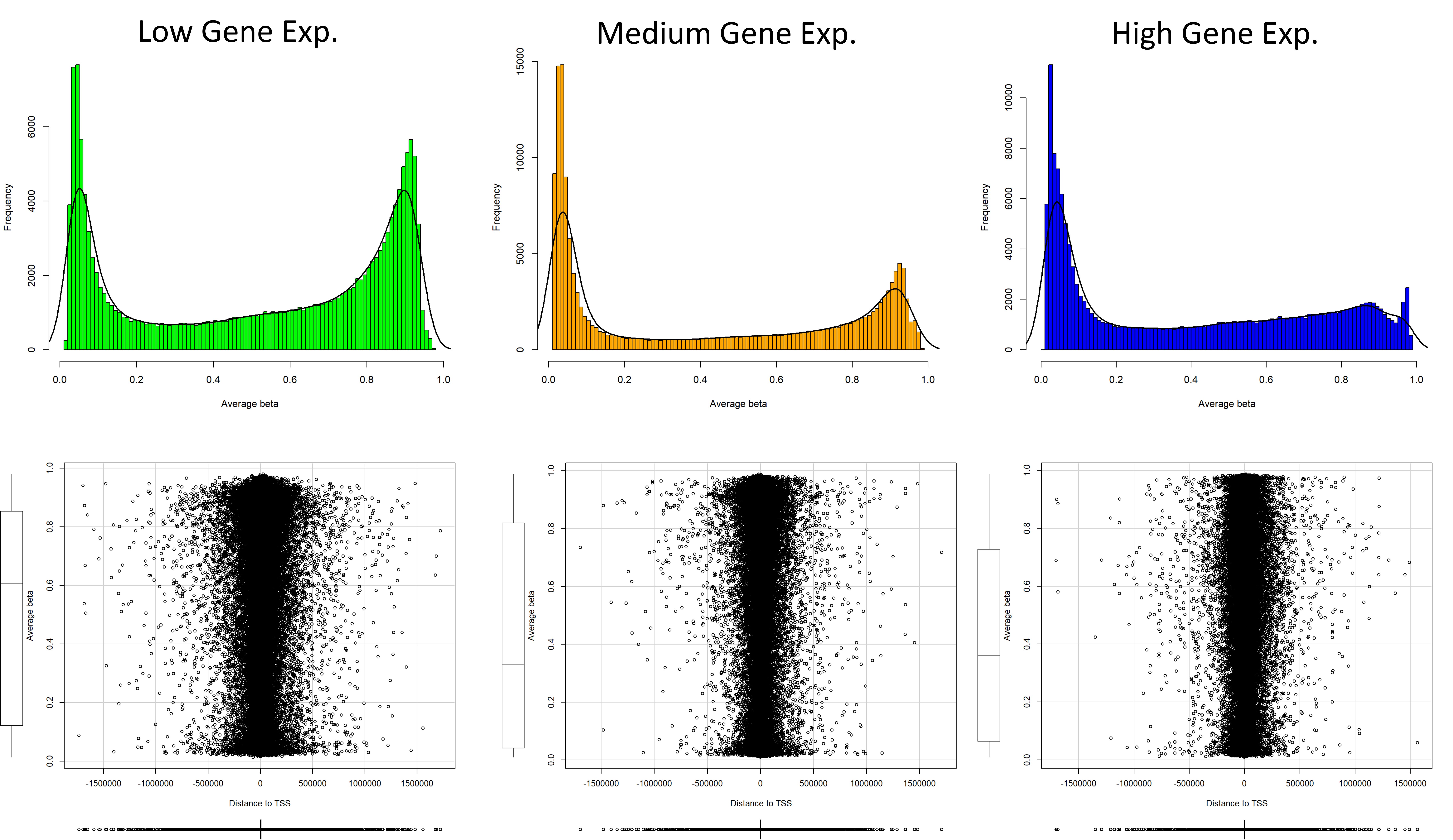

Supplement: Supplementary file 3 [file Image_3.jpg]
